# Neoadjuvant VS adjuvant chemotherapy in patients with locally advanced breast cancer; a retrospective cohort study

**DOI:** 10.1016/j.amsu.2022.104921

**Published:** 2022-11-15

**Authors:** Mohammad-esmaeil Akbari, Mousa Ghelichi-Ghojogh, Zahra Nikeghbalian, Maryam Karami, Atieh Akbari, Mehrdad Hashemi, Saghi Nooraei, Mohsen Ghiasi, Mohammad Fararouei, Farid Moradian

**Affiliations:** a Cancer Research Center, Shahid Beheshti University of Medical Sciences, Tehran, Iran; b Metabolic Disorders Research Center, Golestan University of Medical Science, Gorgan, Iran; c School of Nursing & Midwifery, Shahid Beheshti University of Medical Sciences, Tehran, Iran; d Department of Genetics, Faculty of Advanced Science and Technology, Islamic Azad University, Tehran Medical Sciences, Tehran, Iran; e National Institute for Genetic Engineering and Biotechnology, Tehran, Iran; f HIV/ADIS Research Center, Shiraz University of Medical Science, Shiraz, Iran; g Department of General Surgery, Alborz University of Medical Science, Alborz, Iran

**Keywords:** Breast cancer, Adjuvant, Neoadjuvant, Survival analysis

## Abstract

**Background:**

Breast cancer is one of the most common challenges for women's health. Until now, neoadjuvant chemotherapy is a standard approach in locally advanced breast cancer (LABC), as it increases the probability of breast-conserving surgery (BCS). This study aimed to compare the survival rate in neoadjuvant and adjuvant groups to suggest a better treatment strategy for locally advanced breast cancer.

**Methods:**

The study was conducted between 2009 and 2019 on 845 LABC patients at the Cancer Research Center of Shahid Beheshti University of Medical Sciences in Iran. All patients with LABC at stages 3A, 3B, and two were evaluated for treatment with adjuvants (n = 520 female patients) and neoadjuvant (n = 320 female patients) treatment strategies. Patients were followed up for at least 120 months. The Kaplan-Meier method calculated the survival rate using SPSS version 23 software.

**Result:**

The 5 and 10 years survival rates of neoadjuvant and adjuvant groups were 87 ± 0.04, 80 ± 0.07% and 87 ± 0.02, 83 ± 0.03%, respectively. Statistical analysis results with the mentioned treatment strategies did not show any significant difference in overall survival.

**Conclusion:**

The result of this study on LABC patients demonstrated that compared to surgery first following adjuvant chemotherapy, the neoadjuvant chemotherapy has several benefits, including downstaging and more BCS, with no statistically significant difference in the overall survival rate of the patients.

## Highlights

• The result of this study demonstrated that:
• Although the neo-adjuvant chemotherapy has several benefits and causes down staging and more BCS is possible.
• However it in comparison to surgery first following adjuvant chemotherapy haven't significant statistic difference in overall survival so there is no priority for neo-adjuvant chemotherapy that not only postpone tumor resection but also has many disadvantages such as side effects and alteration of circulatory tumor cells nature.
• Ultimately, it can be stated that if more information is obtained with more samples in the future, it can help confirm the results of this study to provide much more effective results.

## 1 Background

The most common malignancy among women is breast cancer (BC), a worldwide public health concern. In 2018, 9.6 million deaths were reported due to the disease [1,2]. Breast cancer is recognized at the forefront of other cancers in incidence and mortality [2]. Every 3 min, a woman in the United States is affected by breast cancer [3], which is diagnosed in 12% of US women during their lifetime [4]. However, due to the recent advances in early diagnosis and treatment of BC, mortality from the disease has steadily declined since 1990 [5]. The incidence of breast cancer is associated with age, as about 95% of breast cancer cases are reported in women aged 40 years and older [6]. Although the incidence of this type of cancer in Asia is lower than in Western countries, the number of patients is reportedly rising in recent years.

It has been reported that the average age of patients with this cancer in Iran is lower than in other parts of the world [7]. In Iran, the mean age of breast cancer in Iranian women was 47.9 years in 2003, which increased to 49.91 years in 2016 [8], a figure closer to the global average of 58.2 years [9]. Like many other types of cancer, a higher percentage of patients with BC can be treated with a better prognosis if diagnosed earlier [10]. For example, studies have shown that screening for early detection of asymptomatic cases of BC can affect the overall survival rate of the patients [11,12].

Among different types of BC, locally advanced breast cancer (LABC) is a clinically important challenge in cancer treatment. A significant proportion of patients with LABC experience recurrence and death [13]. Adjuvant therapy is an effective treatment strategy that improves outcomes in patients after cancer resection. Applying this approach reduces the risk of cancer recurrence by using systemic chemotherapy and radiation therapy or a combination of both [14].

Neoadjuvant chemotherapy was originally systemic chemotherapy, mainly used for locally advanced breast cancer or inflammatory breast cancer. Recently, however, neo-adjuvant chemotherapy is a combination of chemotherapy, endocrinology, and targeted therapy [15]. This approach is a treatment for breast cancer before surgery to reduce the tumor size, prevent lymph node involvement, and ultimately protect the breast in patients. Neoadjuvant therapy ultimately facilitates surgery and increases the overall survival rate of the patients [16].

Choosing the right treatment for breast cancer is very important because it will significantly impact the patient's quality of life and overall survival [17]. On the other hand, an inappropriate treatment strategy will not only negatively impact the patient's survival and quality of life; it negatively affects their physical, mental, and social health [18]. LABC management predominantly includes neoadjuvant instead of adjuvant chemotherapy, radiotherapy, and other systemic drugs [19]. The neoadjuvant treatment group may have a complete or partial response to treatment, as it may completely eradicate the tumor with preoperative chemotherapy. Studies have shown that breast-conserving surgery with radiation therapy can improve the overall survival and disease-free survival of BC patients [20]. Given the importance of appropriate BC treatment, in recent years, extensive research has been conducted on breast cancer's cellular and molecular mechanisms and the effectiveness of treatment strategies worldwide. This study aimed to compare the survival rate of patients treated according to neo-adjuvant or adjuvant strategies to improve the management of locally advanced breast cancer.

## 2 Methodology

### 2.1 Study design and data collection

All patients diagnosed or treated at the Cancer Research Center of Shahid Beheshti University of Medical Sciences between 2009 and 2019 were studied in this study. The center is the biggest referral center for breast cancer treatment in the capital of Iran (Tehran). In the present investigation, 845 patients with LABC breast cancer were retrospectively studied. In the center, depending on the patient's pathological report and clinical condition, the surgeons decide to follow either adjuvant or neoadjuvant therapy protocol. Surgeries were performed on patients with either breast-conserving surgery or a modified radical mastectomy. All patients in the present study were evaluated through routine follow-up visits and tests, including mammography and ultrasound, for 5–10 years. We compared neoadjuvant and adjuvant groups based on the follow-up period considering different important factors and survival rates. All participants provided written informed consent at the time of their admission. The study was reported in line with the STROCSS guideline [21].

### 2.2 Statistical analysis

In this investigation, the diagnosis date is considered the first event (initial event), and the date of death or the last follow-up is the final event (end-point event). We used Kaplan-Meier to calculate the patients’ survival rate. The log-rank test was used to compare the survival curves. In addition, the Cox model was applied to model the patients' survival rate and its affecting factors. All associations were presented by odds ratio (OR) and 95 confidence interval (CI). The significance level was set at 0.05.

## 3 Results

In this study, a total of 845 patients with BC who were referred to the Tehran Cancer Research Center between 2009 and 2019 and were treated by either adjuvants or neo-adjuvant treatment strategies were evaluated and included in the analysis. Also, all patients who underwent breast-conserving surgery received radiotherapy and a standard medication regimen.

The mean follow-up time for the patients was 120 months. Of 845 patients, 320 were treated with neoadjuvant and 525 with adjuvant methods. The mean age of patients in the neoadjuvant and adjuvant groups was 46 ± 11.4 and 45 ± 17.5 years old, respectively (P = 0.379). During the follow-up period (max = 120 months), 53 deaths occurred in the neoadjuvant group and 38 in the adjuvant group. Moreover, out of 96 recurrences, 27 were from the neoadjuvant group, and 69 were from the adjuvant group. In this study, the number of lymph nodes removed was between zero and 76, with a mean of 13.45 ± 7.1. The tumor size was between 1 and 17 cm with an average of 2.68 ± 2.6 cm. With regard to education, of the neoadjuvant group, 122 patients (38.15%), and of the adjuvant group, 122 patients (23.2%) had diplomas or above (P<0.05%). Also, 207 (64.7%) patients of the neoadjuvant group and 292 (55.6%) patients of the adjuvant group had a family history of breast cancer (P = 0.001). Also, of the neoadjuvant group, 124 patients (38.74%), and of the adjuvant group, 317 patients (60.38%) had positive LVI, respectively (P = 0.001). The distributions of other factors that were examined among the two groups were estrogen receptor (P = 0.003), progesterone receptor (P = 0.048), HER2 (P = 0.009), and hormone therapy (P = 0.036). Data and statistical characteristics of patients for both groups are summarized in Table 1.

**Table 1:** Clinical, demographic and therapeutic characteristics of the studied patients.

Patient information		neoadjuvant	adjuvant	p-value
		Number (percent)	Number (percent)	
Education	Less than a diploma	152(47.5)	262(49.9)	0.001
	Diploma and higher	122(38.1)	122(23.2)	
	Unknown	46(14.4)	141(26.9)	
Marital status	Single	55(17.2)	44(8.4)	0.001
	Married	247(77.2)	385(73.3)	
	Unknown	18(5.6)	96(18.3)	
Family history of cancer	Yes	81(25.4)	111(21.2)	0.001
	No	207(64.7)	292(55.6)	
	Unknown	32(10)	122(23.2)	
History of diabetes	Yes	21(6.6)	39(7.4)	0.001
	No	204(63.8)	250(47.6)	
	Unknown	95(29.7)	236(45)	
Type of surgery	BCS	116(36.3)	317(60.4)	0.001
	MRM	125(39.1)	186(35.4)	
	Unknown	79(24.7)	22(4.2)	
Grade	I	20(6.2)	25(4.8)	0.001
	II	134(41.9)	243(46.3)	
	III	101(31.6)	206(39.2)	
	Unknown	65(20.3)	51(9.7)	
Estrogen receptor	Positive	219(68.4)	347(66.1)	0.003
	Negative	61(19.1)	138(26.3)	
	Unknown	40(12.5)	40(7.6)	
Progesterone receptor	Positive	185(57.8)	320(61)	0.048
	Negative	94(29.4)	164(31.2)	
	Unknown	41(12.8)	41(7.8)	
HER2	Positive	88(27.6)	136(25.9)	0.009
	Negative	181(56.6)	340(64.8)	
	Unknown	51(15.9)	49(9.3)	
Hormone Therapy	Yes	214(66.9)	383(73)	0.036
	No	66(20.6)	102(19.4)	
	Unknown	40(12.5)	40(7.6)	

**Fig. 1:**
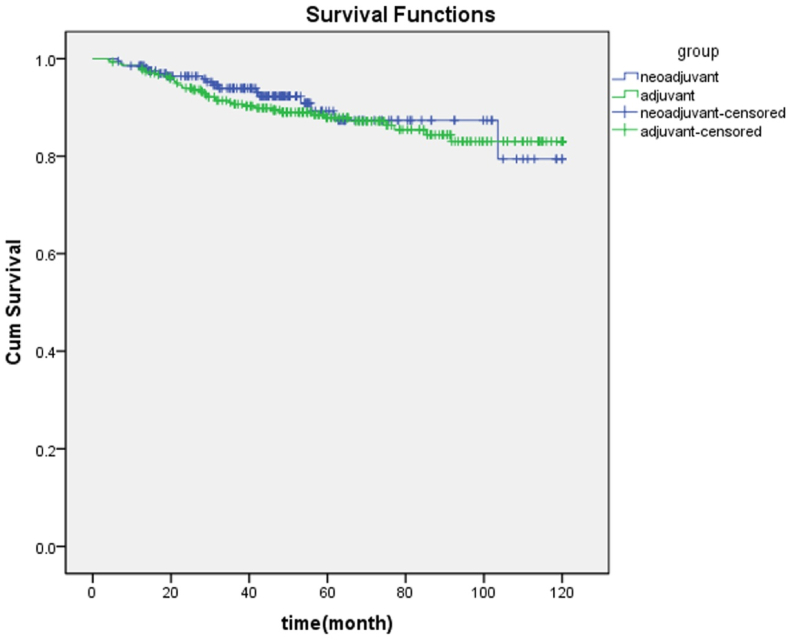
Comparison of survival rate in neo-adjuvant and adjuvant groups.

The overall survival rates of the patients in the neoadjuvant and adjuvant groups evaluated in this study are summarized in Table 2. Accordingly, the mean survival of patients in neoadjuvant and adjuvant groups were 108.22 ± 2.82 and 106.92 ± 1.98 months, respectively. Based on the Log-Rank test results, the difference between the neoadjuvant and adjuvant groups was insignificant (P = 0.542). As shown in Fig. 1, the 5 and 10 years survival rates of the neoadjuvant and adjuvant groups were 87 ± 0.04, 80 ± 0.07% and 87 ± 0.02, 83 ± 0.03%, respectively.

**Table 2:** Comparison of survival rate of patients according to different variables in neo-adjuvant and adjuvant groups.

Variable		Group	Rate of surveillance at year (%)					P-value
			1st year	3rd	5th	7th	10th	
Treatment		Neo-adjuvant	0.01 ± 96	0.02 ± 92	0.04 ± 87	0.04 ± 87	0.07 ± 80	0.542
		Adjuvant	0.01 ± 94	0.02 ± 89	0.02 ± 87	0.03 ± 83	0.03 ± 83	
Education	Academic	Neo-adjuvant	0.01 ± 100	0.04 ± 93	0.04 ± 93	0.04 ± 93	0.04 ± 93	0.585
		Adjuvant	0.03 ± 94	0.03 ± 93	0.04 ± 93	0.05 ± 89	0.03 ± 89	
	Diploma and less	Neo-adjuvant	0.02 ± 94	0.03 ± 91	0.05 ± 83	0.05 ± 83	0.09 ± 75	
		Adjuvant	0.02 ± 94	0.03 ± 89	0.03 ± 87	0.04 ± 82	0.02 ± 82	
Marital status	Married	Neo-adjuvant	0.02 ± 96	0.02 ± 91	0.05 ± 85	0.04 ± 85	0.09 ± 76	0.667
		Adjuvant	0.01 ± 94	0.02 ± 89	0.02 ± 87	0.02 ± 82	0.03 ± 82	
	Single	Neo-adjuvant	0.01 ± 100	0.04 ± 96	0.04 ± 96	0.04 ± 96	0.04 ± 96	
		Adjuvant	0.01 ± 97	0.05 ± 93	0.05 ± 93	0.04 ± 93	0.02 ± 93	
Family history of cancer	YES	Neo-adjuvant	0.02 ± 96	0.02 ± 91	0.05 ± 85	0.04 ± 85	0.09 ± 76	0.601
		Adjuvant	0.03 ± 93	0.04 ± 88	0.04 ± 88	0.07 ± 82	0.07 ± 82	
	NO	Neo-adjuvant	0.02 ± 96	0.02 ± 90	0.05 ± 85	0.04 ± 85	0.04 ± 75	
		Adjuvant	0.01 ± 96	0.02 ± 91	0.03 ± 88	0.04 ± 84	0.04 ± 84	
Type of surgery	MRM	Neo-adjuvant	0.02 ± 95	0.03 ± 90	0.04 ± 88	0.04 ± 88	0.04 ± 88	0.006
		Adjuvant	0.03 ± 91	0.03 ± 89	0.04 ± 85	0.04 ± 83	0.04 ± 83	
	BCS	Neo-adjuvant	0.01 ± 100	0.04 ± 100	0.05 ± 95	0.05 ± 95	0.05 ± 95	
		Adjuvant	0.01 ± 96	0.02 ± 91	0.02 ± 90	0.05 ± 82	0.05 ± 82	
Grade	I	Neo-adjuvant	0.01 ± 100	0.04 ± 88	0.05 ± 88	0.05 ± 88	0.05 ± 88	0.38
		Adjuvant	0.06 ± 94	0.09 ± 87	0.09 ± 87	0.09 ± 87	0.09 ± 87	
	II	Neo-adjuvant	0.03 ± 94	0.03 ± 93	0.04 ± 90	0.04 ± 90	0.04 ± 90	
		Adjuvant	0.03 ± 94	0.03 ± 87	0.03 ± 86	0.04 ± 84	0.04 ± 84	
	III	Neo-adjuvant	0.03 ± 100	0.05 ± 93	0.05 ± 93	0.05 ± 93	0.05 ± 93	
		Adjuvant	0.02 ± 95	0.03 ± 91	0.03 ± 88	0.05 ± 80	0.05 ± 80	
Estrogen receptor	Positive	Neo-adjuvant	0.01 ± 98	0.02 ± 93	0.02 ± 90	0.03 ± 87	0.03 ± 87	0.432
		Adjuvant	0.01 ± 97	0.02 ± 92	0.02 ± 89	0.04 ± 86	0.04 ± 86	
	Negative	Neo-adjuvant	0.03 ± 88	0.04 ± 84	0.04 ± 84	0.05 ± 79	0.05 ± 79	
		Adjuvant	0.04 ± 85	0.05 ± 80	0.03 ± 80	0.06 ± 75	0.06 ± 75	
Progesteronereceptor	Positive	Neo-adjuvant	0.01 ± 98	0.02 ± 94	0.02 ± 92	0.03 ± 86	0.03 ± 86	0.316
		Adjuvant	0.01 ± 97	0.02 ± 92	0.02 ± 90	0.02 ± 84	0.04 ± 84	
	Negative	Neo-adjuvant	0.02 ± 91	0.03 ± 84	0.02 ± 82	0.04 ± 80	0.04 ± 80	
		Adjuvant	0.04 ± 87	0.04 ± 80	0.04 ± 80	0.05 ± 78	0.05 ± 78	
HER-2	Positive	Neo-adjuvant	0.02 ± 97	0.02 ± 94	0.02 ± 94	0.03 ± 94	0.03 ± 94	0.012
		Adjuvant	0.03 ± 95	0.03 ± 92	0.03 ± 92	0.03 ± 92	0.03 ± 92	
	Negative	Neo-adjuvant	0.01 ± 95	0.02 ± 90	0.02 ± 88	0.04 ± 82	0.04 ± 82	
		Adjuvant	0.02 ± 94	0.02 ± 90	0.02 ± 87	0.04 ± 80	0.04 ± 80	
Hormone Therapy	YES	Neo-adjuvant	0.01 ± 96	0.02 ± 92	0.02 ± 89	0.03 ± 86	0.03 ± 86	
		Adjuvant	0.01 ± 95	0.02 ± 91	0.02 ± 88	0.03 ± 84	0.03 ± 84	
	NO	Neo-adjuvant	0.03 ± 91	0.04 ± 86	0.04 ± 86	0.06 ± 81	0.06 ± 81	0.32
		Adjuvant	0.05 ± 86	0.06 ± 80	0.06 ± 80	0.08 ± 75	0.08 ± 75	
History of Diabetes	YES	Neo-adjuvant	0.02 ± 100	0.05 ± 93	0.06 ± 89	0.09 ± 82	0.09 ± 82	0.001
		Adjuvant	0.02 ± 100	0.06 ± 91	0.06 ± 91	0.1 ± 83	0.1 ± 83	
	NO	Neo-adjuvant	0.01 ± 97	0.01 ± 95	0.02 ± 94	0.04 ± 90	0.04 ± 90	
		Adjuvant	0.01 ± 97	0.01 ± 94	0.02 ± 93	0.04 ± 88	0.04 ± 88	
LVI	Positive	Neo-adjuvant	0.01 ± 99	0.04 ± 90	0.04 ± 90	0.09 ± 89	0.09 ± 88	
		Adjuvant	0.01 ± 98	0.04 ± 87	0.03 ± 85	0.04 ± 82	0.04 ± 82	
	Negative	Neo-adjuvant	0.01 ± 95	0.02 ± 90	0.02 ± 88	0.04 ± 82	0.04 ± 82	
		Adjuvant	100	0.03 ± 94	0.02 ± 94	0.03 ± 85	0.03 ± 85

Cox regression was used to model factors affecting patients' survival, and the results are presented in Table 3. Accordingly, a patient's age was an influential factor in her/his mortality, so the risk of death in patients over 50 years old was 1.36 times higher than in those younger (P = 0.032).

**Table 3:** Crude and adjusted associations between the study variables and breast cancer mortality (Regression COX).

Variable		Univariate			Multivariable		
		HR	95%CI	P value	HR	95%CI	P value
Age (year)	<50	1^a^	–	–	1^a^	–	–
	>50	1.42	1.14–2.31	0.04	1.36	1.11–2.13	0.032
Education	Academic	1^a^	–	–	1^a^	–	–
	Diploma and less	1.86	1.23–2.88	0.003	2.04	1.15–3.62	0.001
Marital status	Single	1^a^	–	–	–	–	–
	Married	0.62	0.346–1.04	0.35	–	–	–
Estrogen receptor	Positive	1^a^	–	–	1^a^	–	–
	Negative	2.19	1.31–3.76	0.001	2.06	1.17–3.66	0.001
HER-2	Positive	1^a^	–	–	–	–	–
	Negative	1.45	0.8–2.75	0.45	–	–	–
Type of surgery	MRM	1^a^	–	–	–	–	–
	BCS	0.86	0.46–1.34	0.65	–	–	–
Grade	1	1^a^	–	–	1^a^	–	–
	2	1.86	1.21–3.42	0.012	1.74	1.19–3.12	0.001
	3	2.38	1.61–5.83	0.01	2.22	1.58–5.68	0.001
Family history of cancer	No	1^a^	–	–	1^a^	–	–
	Yes	3.17	1.91–5.26	0.007	2.22	1.23–4.88	0.004

a = **Reference category**.

The grade of the tumor was also identified as one of the important contributing factors in the mortality of the patients. Accordingly, the risk of death among grade II and III patients was 1.86 times and 2.38 times higher than those with grade I (P < 0.05). Moreover, a family history of breast cancer was also identified as one of the most influential factors in the mortality rate of patients (the risk of death among those with a family history of breast cancer was 3.17 times higher than patients with no such history) (P = 0.004).

**Fig. 2:**
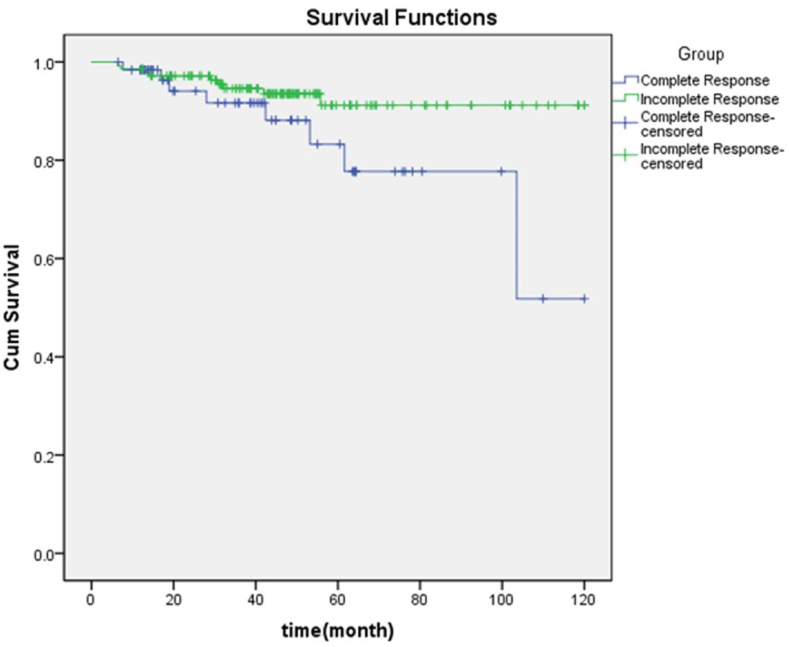
Comparison of survival rate in complete response and incomplete response groups in neo-adjuvant patients.

In our study, among the neoadjuvant patients, the group with a complete response was compared with the group with a partial response to neoadjuvant in terms of survival and relapse. According to Fig. 2, the 1, 3, 5, 7, and 10 years survival rates of patients in complete response were 94 ± 0.03, 89 ± 0.05, 78 ± 0.09, 78 ± 0.09, and 55 ± 0.02, respectively. While in the incomplete response group, the rates were 97 ± 0.01, 93 ± 0.02, 92 ± 0.02, 92 ± 0.02, and 92 ± 0.03 respectively. The mean survival time was 98.07 ± 6.7 months for complete response and 112.26 ± 2.57 months for incomplete response (P = 0.032).

Of the 27 recurrent cases of cancer that occurred in neoadjuvant patients, 8 patients (29.6%) were in the complete response group, and 19 (70.4%) were in the incomplete response group (P = 0.558).

**Fig. 3:**
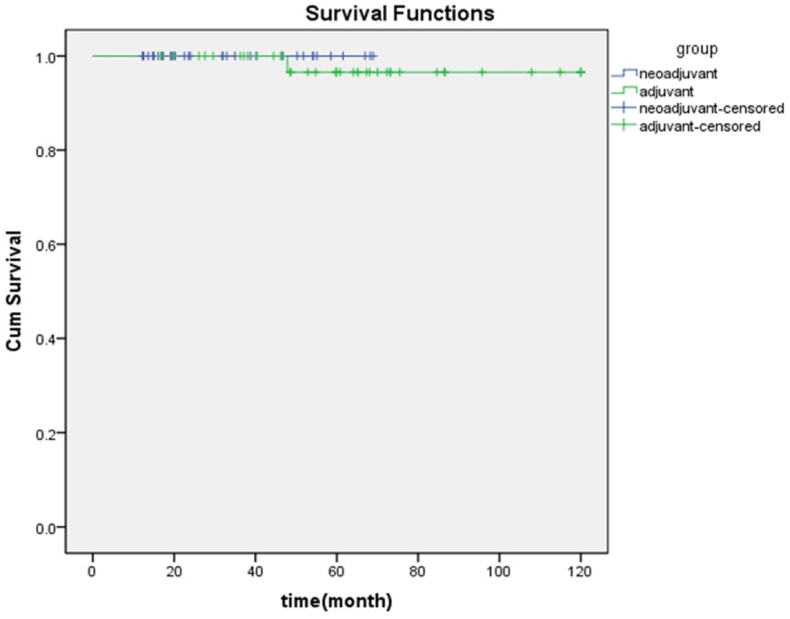
Comparison of survival rates in Neo-adjuvant and Adjuvant positive receptor groups (ER, PR and HER2 positive).

The analysis showed that out of 320 neoadjuvant patients, 50 (15.62%) patients were positive for ER, PR, and HER2. Almost similarly (P > 0.05), in the adjuvant group, 64 (12.19%) patients were positive for all three receptors. According to Fig. 3, One, three, and five years survival rates of patients in the neoadjuvant group with all positive receptors (ER, PR, and HER2 positive) were 100, 100, and 100%, respectively, and in the adjuvant group were 100%, 97%, and 97% respectively. This difference was not significant according to the Log-Rank test (P = 0.557).

The results of our study showed that out of 320 neoadjuvant patients, 31 (9.68%) were ER, PR, and HER2 negative, and of 525 adjuvant patients, 70 (13.3%) were negative for all three receptors (P > 0.05). According to Supplementary Fig. 1, the 1, 3, and 5 years survival rates of in neoadjuvant group with all negative receptors (ER, PR, and HER2 negative) were 89 ± 0.08, 77 ± 0.13, and 77 ± 0.13%, respectively, and in the adjuvant group were 82 ± 0.06, 73 ± 0.08, and 73 ± 0.08% respectively. Again, this difference was not significant according to the Log-Rank test (P = 0.584).

In addition, the 1, 3, 5, 7, and 10 years survival rates of patients with MRM in the complete response group were 100, 100, 100, 100, and 100%, respectively, and in the incomplete response group, were 94, 89, 86, and 86 respectively (Supplementary Fig. 2). Also, the 1, 3, 5, 7, and 10 years survival rates of patients with BCS in the complete response group were 100%, 100%, 100%, 100%, and 100%, respectively, and in the incomplete response, group were 100%, 100%, 100%, 100%, and 100% respectively (Supplementary Fig. 3).

## 4 Discussion

Many women with early-stage cancer can choose between BCS and mastectomy [22]. The main advantage of BCS is that patients can have their breasts. However, in most cases, they also need radiation therapy, and women who undergo mastectomy for early-stage cancer need less radiation therapy. In addition, for some women, mastectomy is a better option because of the type of cancer, the size of the tumor, previous radiation treatment, or other factors [22].

In the present study, several patients underwent neoadjuvant therapy. These patients were either LABCs, had axillary involvement, had a large tumor size, had affected skin, or wanted to maintain their breasts. Also, if the surgeon was uncomfortable with operating and felt that a large mass was attached to the underlying tissue or the patient might face bleeding, the neoadjuvant strategy was applied.

The results obtained in this study indicated that education, hormone therapy, estrogen and progesterone receptor status, and tumor grade were not significantly affecting the overall survival of neoadjuvant and adjuvant groups. However, factors such as family history, HER-2 receptor status, MRM or BCS surgery, history of diabetes, presence or absence of LVI, and patient's age had significant effects on the overall survival of the patients. The treatment strategy (adjuvant or neo-adjuvant methods) did not affect the survival of the patients. Currently, two adjuvants and neoadjuvant strategies exist for treating patients with LABC. Breast preservation is feasible after neoadjuvant chemotherapy in many patients where surgery is possible. The neoadjuvant strategy can increase the amount of conserved breast surgery in patients but does not significantly increase their survival [23]. This method is a beneficial feature because it does not cause a negative feeling about the patient's body image. Adjuvant strategy, on the other hand, in addition to the negative psychological effects, can also cause adverse physical events [23]. In this treatment strategy, the proper selection of patients and the appropriate drug combination with the appropriate dose over a specified period are crucial. This choice should be carefully considered to avoid drug resistance, and it should be noted that this method may not have the same effect on all patients. In neoadjuvant chemotherapy, it is possible to create secondary resistance to treatment after chemotherapy. Post neoadjuvant surgery is also a challenge especially in the complete response cases of LABC. The strategy is used for advanced stages of BC [[24], [25], [26], [27]]. Some scientific reports worldwide reported a significant enhancement in the overall survival of LABC patients treated with a neoadjuvant strategy [26,28]. It has been mentioned in many articles that the neoadjuvant technique can reduce tumor size, migration of cancer cells to other tissues, and tumor margins. However, the various aspects of this strategy haven't been well defined yet [29]. In addition, our study showed that surgery in LABC patients and then chemotherapy with the adjuvant method compared to the neoadjuvant chemotherapy did not indicate any significant difference in the overall survival of the patients. Given that in recent years, there has been controversy over the choice of neoadjuvant and adjuvant, our results may carry out an important role in choosing the treatment strategy for the patients. The results of this study indicated that the neoadjuvant strategy isn't a priority. Given that most previous studies have been performed by oncoradiotherapists and in most studies, less attention has been paid to the molecular aspects, we could argue that the study of cellular and molecular pathways and their role in response to chemotherapy as well as cancer stem cells are immensely important missing aspects of BC. Given the results of this study, it is likely that the role of secondary chemotherapy resistance in neoadjuvant treatment is frequently overlooked. From a molecular perspective, chemotherapy resistance and metastatic variable phenotype are the most important causes of failure in the treatment of breast cancer and cancer-related mortality. Recently, a series of experimental and clinical studies have pointed to the key role of stem cells in the chemical resistance and metastasis of cancer. Research shows that breast cancer is caused by a small number of cancer cells called cancer stem cells or tumor stem cells. Cancer stem cells (CSCs) have unlimited regenerative and proliferative power [[30], [31], [32]]. In that regard, CSCs, as well as natural stem cells, possess several properties, such as drug resistance or toxins through the expression of their drug-specific transporter, ability to repair DNA, and resistance to apoptosis and hypoxia, factors that are critical for long-term survival. As a result, conventional radiotherapy and chemotherapy can only kill the bulk of the tumor, not the cancer stem cells, and these cells will survive over time and develop into a new tumor [33,34]. Several growth pathways, such as Notch, Sonic Hedgehog, and WNT, regulate the self-renewal of natural stem cells. Many stem cell-related genes are oncogenic, and many genes that inhibit self-renewal are also tumor suppressor genes [35]. Observations suggest that CSCs originate from natural stem cells by accumulating somatic mutations during aging. The present compounds in the intrinsic pathways of the cell may override the nature of CSCs, thereby preventing the development of CSCs clones that lead to tumor initiation or even tumor recurrence. Inhibition of the WNT pathway can effectively prevent the recurrence of CSCs [[35], [36], [37]]. Treatment failure is partly due to the heterogeneity of phenotypic variation in CSCs. Each cancer subgroup contains distinct CSCs subtypes that express different CSC markers, and the molecular differences of CSCs indicate various outcomes in response to the current therapies. The evidence presented so far illustrates that using neo-adjuvant treatment can affect cancer stem cells. As a result, treatment becomes more difficult. This may be why people treated with neoadjuvant treatment do not see a significant difference in their recovery compared to those treated with adjuvant treatment. It should be noted that the breast cancer stromal compartment may also affect the response to chemotherapy. The behavior of BC is a reflection of the signal interaction between breast epithelial cells and the surrounding microenvironment including cancer-associated fibroblasts, mesenchymal stem cells, tumor-associated macrophages, endothelial cells, lymphocyte cells, and adipose as well as matrix components extracellular [38]. There is some evidence that normal tissue stromal cells have a significant difference with tumor cell stromal cells, which is significantly related to the expression of gene profiles. Moreover, there is evidence that extracellular matrix, such as stromal cells, may play a role in the response to treatment and drug resistance. It has been suggested that overexpression of some genes in the stroma, which is a stromal response to neo-adjuvant therapy, may also produce secondary resistance to the treatment by affecting estrogen receptors [38]. Studies have identified several genes that contribute to the secondary resistance to treatment including SH2D1A, TOY, and LY75 genes. In addition, GATA3, ERBB4, RET, NAT, and TFF3 genes are commonly expressed in tumor cells, which may also influence response to treatment. Also, It seems that the AR gene resists therapy in neoadjuvant therapy. Studies have shown that KEGG and ECM cross-pathways play an important role in causing this resistance [[39], [40], [41]].

Advantages and limitations: Our study involves a significant number of patients and a relatively long follow-up period. We also applied several important clinical and pathologic factors in our analysis. However, due to ethical considerations, the surgeons did not randomly select the treatment strategy for each patient. This may cause some important differences between the two study groups with regard to factors affecting the survival of BC patients. We tried to control for such errors in our study by applying a multivariate approach to data analysis.

## 5 Conclusion

In conclusion, the result of this study on LABC patients demonstrated that although neoadjuvant chemotherapy has several benefits and causes downstaging and more BCS, in comparison to surgery first following adjuvant chemotherapy, haven't significant benefit in the overall survival of the patients. As a result, there is no priori for neoadjuvant chemotherapy in postponing tumor resection, and also it has many disadvantages such as side effects and alteration of circulatory tumor cells nature. Ultimately, it can be stated that if more information is obtained via randomized interventions in the future, we may be able to confirm the results of this study to provide much more effective treatment strategies.

## Ethical approval

An informed consent was obtained from all participants. The analysis data file did not contain the name of the participants. This study was approved by the research ethics committee of Shahid Beheshti University of Medical Sciences (IR.SUMS.CRC.1398.019).

## Sources of funding

This study was approved and financially supported by 10.13039/501100004320Shiraz University of Medical Sciences (Grant number: 16976).

## Author contribution

All authors contributed to the study conception and design. MSA and MF participated in the design of the study. ZN, MK and SN performed data collection and wrote the manuscript. AA and MH revised the manuscript. MGG and MF helped with statistical analysis and prepared the illustrations. MG and FM edited the manuscript. All authors read and approved the final manuscript.

## Registration of research

1 Name of the registry:
2 Unique Identifying number or registration ID:
3 Hyperlink to your specific registration (must be publicly accessible and will be checked):

## Guarantor

*Corresponding **author**Farid Moradian: Department of General Surgery, Alborz University of Medical Science, Alborz, Iran. Tel: (98) 212 2724204; Fax: (98) 212 274800E-mail: moradian.farid@yahoo.comORCID: 0000-0001-9651.

## Availability of data and materials

The datasets used and/or analyzed during the current study are available from the corresponding author on reasonable request.

## Provenance and peer review

Not commissioned, externally peer-reviewed.

## Abbreviations

LABC: locally advanced breast cancer, BCS: Breast-conserving surgery, MRM: Modified radical mastectomy, OR: odds ratio, CI: confidence interval, CSC: Cancer stem cells.

## Consent

An informed consent was obtained from all participants. The analysis data file did not contain the name of the participants.

## Declaration of competing interest

The authors declare that they have no **conflicts** of interests.
