# Supplementary material for: Neoadjuvant VS adjuvant chemotherapy in patients with locally advanced breast cancer; a retrospective cohort study
Source: Ann Med Surg (Lond). 2022 Nov 15;84:104921. doi: 10.1016/j.amsu.2022.104921 (PMC9758373; doi:10.1016/j.amsu.2022.104921)
Supplement: Multimedia component 1 [file mmc1.docx]

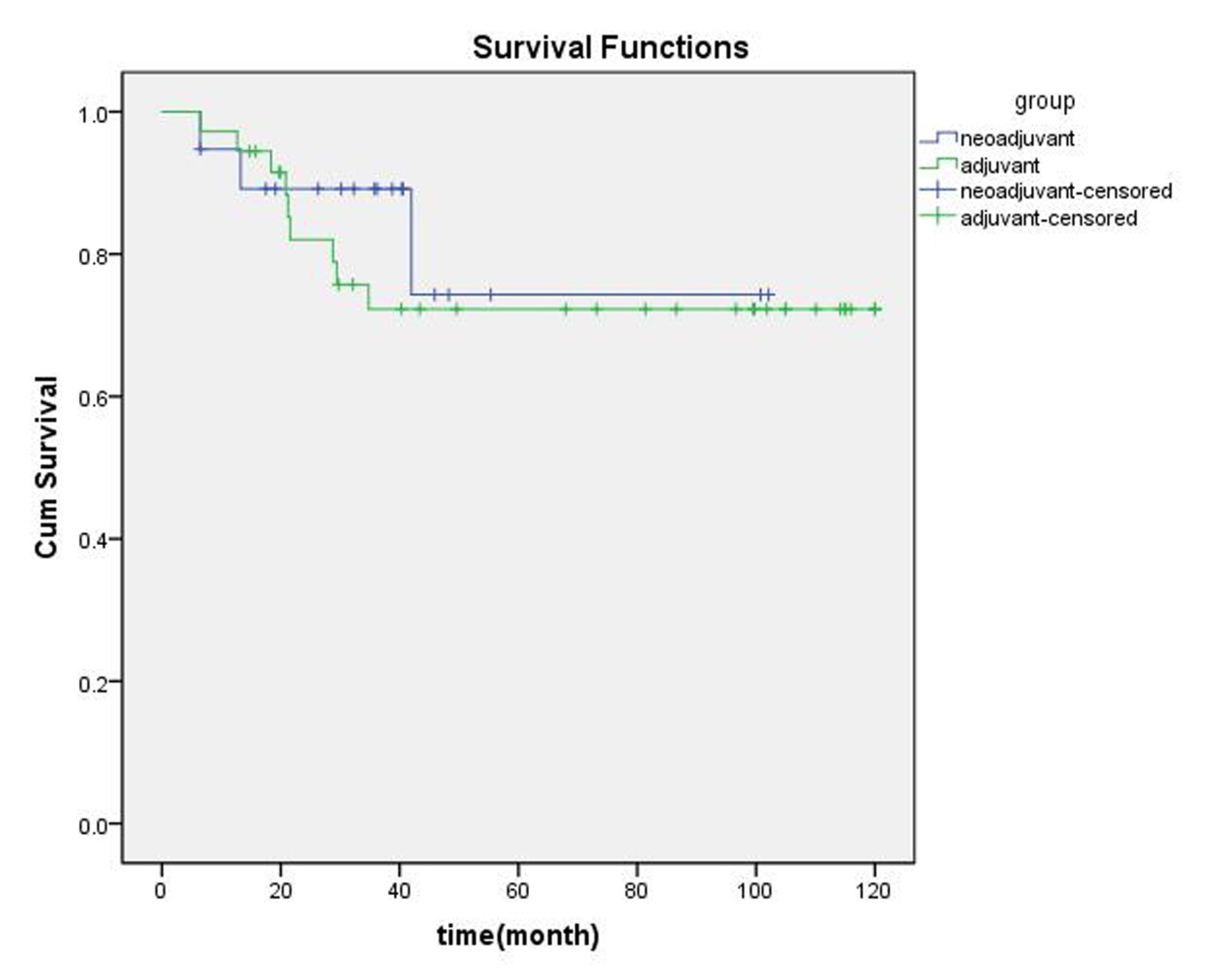


Supplementary Figure 1. Comparison of survival rate in neo-adjuvant and adjuvant negative receptor groups (ER, PR and HER2 negative)
